# Exploring the mechanism of triptolide inhibiting the motility of fibroblast-like synoviocytes in rheumatoid arthritis via RhoA/Rho-associated kinase axis, based on network pharmacology, molecular docking and molecular dynamics simulations

**DOI:** 10.3389/fphar.2025.1545514

**Published:** 2025-04-03

**Authors:** Jiacheng Shen, Yuxuan Fang, Nan Xu, Hongyi Chen, Miao Zhu, Dan Li, Zewen Chu, Masataka Sunagawa, Yanqing Liu, Haibo Wang, Guoqing Li

**Affiliations:** ^1^ Department of Rheumatology and Immunology, Affiliated Hospital of Yangzhou University, Yangzhou University, Yangzhou, Jiangsu, China; ^2^ Medical College, Institute of Translational Medicine, Yangzhou University, Yangzhou, China; ^3^ The Key Laboratory of Syndrome Differentiation and Treatment of Gastric Cancer of the State Administration of Traditional Chinese Medicine, Yangzhou, China; ^4^ School of Rehabilitation Science, Shanghai University of Traditional Chinese Medicine, Shanghai, China; ^5^ Department of Physiology, School of Medicine, Showa University, Tokyo, Japan

**Keywords:** rheumatoid arthritis, triptolide, cytoskeleton, RhoA/Rho-associated kinase, network pharmacology, molecular dynamics simulations

## Abstract

**Background and objective:**

Rheumatoid arthritis (RA) is a chronic autoimmune disease characterized by the hyperproliferation and invasive behavior of rheumatoid arthritis fibroblast-like synoviocytes (RA-FLS), which contributes to the degradation of articular cartilage and bone. Inhibition of RA-FLS proliferation, migration and invasion has become an important therapeutic strategy for RA. Triptolide (TPL), an epoxy diterpene lactone compound from the traditional Chinese medicine *Tripterygium wilfordii Hook. f.*, has significant immunosuppressive and anti-inflammatory effects. However, the specific mechanisms of TPL-regulated effects on RA-FLS cytoskeleton and inhibition of invasive metastasis are not yet fully explored. The aim of this study was to investigate TPL-regulated effects on RA-FLS skeleton and reveal the specific mechanism of TPL-inhibition of RA-FLS migration and invasion.

**Materials and methods:**

*In vitro* experiments were performed using RA-FLS cell line. Cell motility was evaluated by wound healing assay and Transwell assay as well as high content cell imaging system. Cytoskeletal remodeling was observed by cytoskeletal immunofluorescence staining and transmission electron microscopy (TEM). Network pharmacology predicted the targets of Triptolide. RhoA/Rho-associated kinase signaling pathway was detected by quantitative real-time PCR and Western blotting. Molecular docking and molecular dynamics simulations were used to validate the interaction of Triptolide with RhoA/Rho-associated kinase.

**Results:**

TPL significantly inhibited RA-FLS cell motility, and reduced the displacement and cumulative distance of RA-FLS. Cytoskeleton staining assay and TEM observation showed cytoskeleton remodeling after TPL treatment. Network pharmacological prediction screened 45 targets associated with TPL intervention in RA via cytoskeleton, including TNF, KRAS, ESR1, RHOA, MAPK3 and CASP3. In the RhoA/Rho-associated kinase signaling pathway, TPL treatment inhibited protein expression and phosphorylation of RhoA, Rock, and Limk. TPL can enter RhoA, Rock1, and Rock2 target protein binding domains with stable binding activities, and may cause conformational changes of Rock1 related to molecular functions.

**Conclusion:**

TPL inhibits RA-FLS in motility by regulating actin cytoskeleton remodeling through action on the RhoA/Rho-associated kinase signaling pathway.

## Graphical Abstract

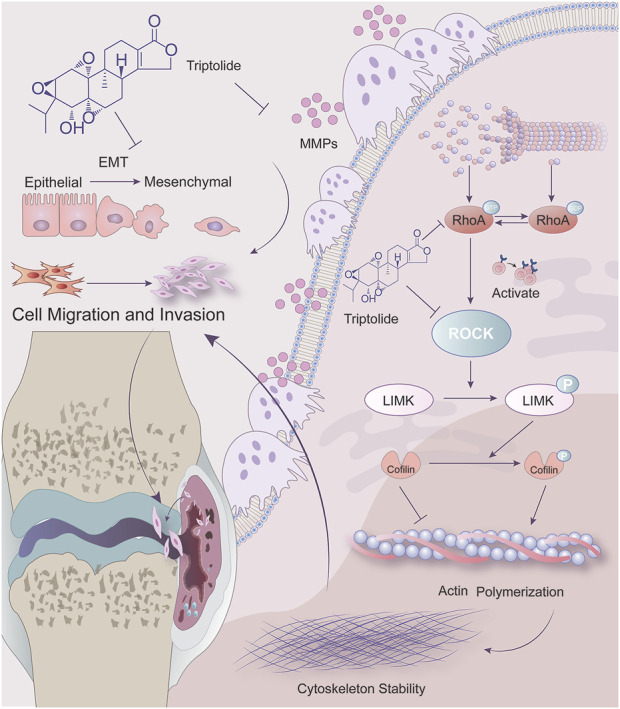

## 1 Introduction

Rheumatoid arthritis (RA) is a systemic autoimmune disease characterized by progressive joint destruction. Epidemiologic surveys reveal a global incidence of 0.5%–1%, and a prevalence of 0.42% in mainland China, with significantly higher incidence in females than males (female-to-male ratio of 4:1) (Zhonghua, 2024). At the pathological level, the invasive pannus formed by abnormal synovial hyperplasia constitutes the core mechanism of joint destruction, where RA fibroblast-like synoviocytes (RA-FLSs) exhibit tumor-like biological behaviors - their migratory capacity and matrix invasiveness fundamentally differ from normal synoviocytes (Gangishetti et al., 2020). These activated RA-FLSs accelerate joint damage through multiple pathways: releasing inflammatory cytokines (e.g., TNF-α, IL-1, IL-6), secreting matrix metalloproteinases to degrade cartilage, and directly eroding bone tissue via osteoclast differentiation regulation (Singh et al., 2023; Yoshitomi, 2019). Notably, untreated patients may develop irreversible joint structural damage within short periods, underscoring the critical clinical significance of elucidating RA-FLS invasion mechanisms.

Recent studies demonstrate that unlike physiological synoviocytes, RA-FLSs acquire sustained migratory capacity through activation of Rho GTPase signaling, with the RhoA/ROCK axis playing a particularly crucial role. Under hypoxic conditions, the HIF-1α-dependent RhoA pathway significantly contributes to cytoskeletal remodeling and RA-FLS migration (Chen J. et al., 2023). ROCK inhibitors have been demonstrated to ameliorate RA in the K/BxN serum transfer arthritis mouse model through dual mechanisms of attenuating inflammation and suppressing bone erosion (Rodríguez-Trillo et al., 2022). The RhoA/ROCK signaling pathway precisely regulates invasive migration of RA-FLSs. RhoA activates downstream effector ROCK to drive myosin light chain (MLC) phosphorylation, facilitating actin-myosin filament assembly and forming transcellular stress fiber networks - these contractile fibers provide mechanical force for matrix barrier penetration (Singh et al., 2021; Yu et al., 2020). ROCK additionally establishes localized actin polymerization dominance at the cell front by phosphorylating LIM kinase to indirectly inhibit actin-depolymerizing factors (e.g., cofilin), thereby promoting directional extension of invasive pseudopodia (Roy et al., 2024). This spatiotemporally coordinated regulation enables RA-FLSs to breach synovial barriers and initiate pathological joint erosion. Emerging evidence reveals a positive feedback loop between RhoA/ROCK signaling and Wnt5a pathway in RA-FLSs, where Wnt5a further amplifies RhoA activity through non-canonical pathways, sustaining overexpression of invasion-related genes MMP-3/9 (Rodriguez-Trillo et al., 2020). Nevertheless, systematic understanding of RhoA/ROCK-mediated cytoskeletal regulatory networks remains incomplete, particularly regarding their dynamic remodeling under pharmacological intervention.

Triptolide (TPL), a natural epoxy diterpene lactone compound primarily extracted from the roots of Tripterygium wilfordii Hook. f., exhibits unique anti-RA properties. Previous research predominantly focused on its anti-inflammatory characteristics, including inhibition of pro-inflammatory cytokines (e.g., IL-6, TNF-α) (Piao et al., 2021; Zhang et al., 2019) and induction of RA-FLS apoptosis (Lin et al., 2021). Recent investigations demonstrate that TPL can induce apoptosis in MDA-MB-231 breast cancer cells by modulating RhoA and ROCK1 expression, while suppressing epithelial-mesenchymal transition, proliferation, and invasion (Liu et al., 2019a; Wu et al., 2023). These findings suggest the RhoA/ROCK signaling pathway may serve as a potential therapeutic target for TPL’s anti-invasive effects, possibly through regulation of cytoskeletal dynamics in RA-FLSs. However, the precise mechanistic basis of this action remains to be fully elucidated.

## 2 Experimental

### 2.1 Drugs and regents

Triptolide (standard, HPLC ≥99.86%) was purchased from MedChemExpress LLC (Cat. No. HY-32735). Triptolide was dissolved in dimethyl sulfoxide (DMSO, Sigma, Berlin, Germany, Cat. No. D2650). The maximum concentration of DMSO in the medium should not exceed 0.1%. All control groups received an equal volume of DMSO in DMEM medium, equivalent to the highest Triptolide dose.

DMEM medium (Gibco, China, Cat. No. C11995500BT); fetal bovine serum (FBS; Gibco, Brazil, Cat. No. A5256701); penicillin-streptomycin solution (Solarbio Science and Technology, Beijing, China Cat. No. P1400); 6.5 mm Transwell 8.0 µm pore size polycarbonate membrane chambers (Corning, New York, United States, Cat. No. 3422); Matrigel basement membranes (Corning, New York, United States, Cat. No. 356234); Tumour Necrosis Factor alpha (TNF-α, MedChemExpress LLC, Shanghai, China, Cat. No. HY-P7058); phalloidin- Alexa Fluor 555, an actin tracker (Beyotime Biotechnology, Shanghai, China, Cat. No. C2203S). The relevant primer sequences for quantitative real-time PCR listed in Supplementary Table S1 are customized and ordered from Sangon Biotech (Shanghai) Co., Ltd. The antibodies used for Western blotting in this study are listed in Supplementary Table S2.

### 2.2 Cell culture

Rheumatoid arthritis human fibroblast-like synoviocytes (RA-FLS) cell line MH7A and human fibroblast-like synoviocytes (HFLS) were purchased from Guangzhou Jenniobio Biotechnology Co., Ltd. (Guangzhou, China) and cultured in DMEM medium, containing 10% fetal bovine serum and 1% penicillin-streptomycin solution. The cells were cultured at 37°C in a 5% CO_2_ humidified atmosphere.

### 2.3 Cell migration and invasion assay

The cell migration and invasion capabilities were assessed using wound healing assay and Transwell chambers, respectively. Cells were seeded in the upper chambers (coated with matrigel) at a density of 1 × 10^5^ cells/mL in serum-free medium and the lower chambers contained 10% FBS medium with different TPL concentrations (0, 20, 40, and 80 nM). Following a 24-h incubation period, cells on the upper membrane were removed, and those that had invaded the lower chamber were fixed with 4% polyformaldehyde and stained with crystal violet. Images were taken using a microscope (Olympus, Japan) at ×100 magnification.

### 2.4 High-content cell imaging system

The samples, containing 4,000–8,000 per well, were transferred to the PerkinElmer Operetta CLS high-content imaging system (PerkinElmer) after 24 h of incubation at 37°C. The dissection level was adjusted based on bright-field images, and the cell motility detection module was selected. The system was programmed with temperature settings, recording intervals, and culture conditions. Cells were then continuously monitored for an additional 12 h, during which the high-content imaging system automatically tracked cell division, proliferation, and motility. Data collection and analysis were performed using Harmony software (PerkinElmer).

### 2.5 Immunofluorescence staining of cytoskeleton

A sterile glass slide was positioned at the bottom of a 6-well plate. RA-FLS cells were evenly inoculated into each well and treated with medicated medium for 24 h. Cells on glass slides were then fixed with 4% polyformaldehyde and permeabilized with 0.1% Triton X-100. To stain the cytoskeleton protein, Phalloidin-Alexa Fluor 555 was added and then incubated at room temperature in the dark for 1 h. The slides were mounted with an anti-fluorescence quenching mounting medium after incubating with the DAPI staining solution. The cytoskeleton morphology was observed and recorded using a laser confocal microscope.

### 2.6 Transmission electron microscopy (TEM)

To visualize cytoskeletal remodeling, we used transmission electron microscopy to observe microfilament and microtubule distribution within cells. After removing the medium from drug-treated cells (cell density ≤70%), trypsin was added to digest the cells. The cell sediment was collected by gentle aspiration and centrifuged for 3–5 min at low speed. Cell clumps should be of similar size to beans. The supernatant was discarded, and the cells were dispersed and resuspended with electron microscope fixative (Wuhan Safeway Biotechnology Co., Ltd.) at room temperature. The cells should be fixed for 30 min at room temperature, protected from light, and then transferred to 4°C for storage. TEM experiments were conducted by Wuhan Servicebio Biotechnology Co., Ltd. Images were acquired using a TEM (Hitachi, Ltd., Tokyo, Japan).

### 2.7 Quantitative real-time PCR

The RNA Easy Fast Animal Tissue/Cell Total RNA Extraction Kit (Tiangen Biotechnology (Beijing) Co.) was utilized for RNA extraction. In brief, 3–5 × 10^6 cells were collected, treated with 350 μL of lysis buffer, 10 μL of Proteinase K added. After thorough mixing and 5 min incubation at room temperature, RNA was extracted following subsequent steps, and its concentration determined. For cDNA synthesis by reverse transcription of total RNA, 1 μg of total RNA sample was added to an ice-bath RNase -free PCR tube with 4 μL of 5×PrimeScript Mix, topped up with RNase-free water to 20 μL. The reaction mixture was transferred to a PCR machine and set to 37°C for 15 min, 85°C for 5 s, and 4°C forever. Fluorescent quantitative PCR amplification (Roche, Basel, Switzerland) was performed with initial denaturation at 95°C for 600 s, followed by 95°C for 10 s, 55°C for 10 s, and 72°C for 10 s for 45 cycles, and a final step of 95°C for 10 s, 65°C for 60 s, and 97°C for 1 s. Results were recorded, and data processed and analyzed.

### 2.8 Network pharmacology

Target prediction screening was performed using CHEMBL (https://www.ebi.ac.uk/chembl/) and searching relevant literature (Liu et al., 2019b); (Wang et al., 2016; Liu et al., 2013), and targets with P > 0 were selected as potential targets for Triptolide. The results from these databases were integrated and de-emphasized, and the target names were standardized using the Uniprot database (https://www.uniprot.org/) to ensure completeness of the compound targets. The keywords “rheumatoid arthritis” and “cytoskeleton” were used to search for relevant targets in the Genecards (https://www.genecards.org/) database. The obtained target names were imported into the Uniprot database for normalization, and the results of these databases were integrated and de-emphasized to obtain the targets for the relevant diseases. Venny2.1.0 (https://bioinfogp.cnb.csic.es/tools/venny/) was used to draw the Venn diagram to obtain the intersecting targets. Corresponding to the compounds and intersection targets, we created “Network” and “Type” files, and used Cytoscape 3.9.1 to construct the network diagram of compounds and intersection targets, and used topological analysis to obtain the relevant data of the network diagram. We used String database (https://cn.string-db.org/) to analyze protein interactions of the intersecting targets, with an interaction factor of 0.4 to hide free targets, and exported network information to “TSV” file. Cytoscape 3.9.1 was used to open the “TSV” file, construct the PPI network, and obtain data related to the network diagram through topological analysis. The David database (https://david.ncifcrf.gov/) was used for enrichment analysis of intersecting targets, with visualization performed using microbiological letters. The pathways to be visualized and their enriched targets corresponded with “Network” and “Type” files created. Cytoscape 3.9.1 was used to construct the compound-target-pathway network diagram, and topological analysis used to obtain relevant data. Topological analysis obtained relevant data of the network diagram.

### 2.9 Molecular docking

The 2D structure of Triptolide (CID_107,985) was retrieved from the PubChem database (http://pubchem.ncbi.nlm.nih.gov/) and used as input to generate its 3D structure using Chem Office 20.0 software. The RCSB PDB database (http://www.rcsb.org/) was utilized to screen high-resolution crystal structures of the protein target Rhoa (PDB: 1CXZ), Rock1(PDB: 2ETR), and Rock2 (PDB: 4L6Q) as potential molecular pair acceptors. PyMOL 2.6.0 was utilized to dehydrate and dephosphorylate the proteins. The Molecular Operating Environment (MOE) 2019 was utilized to minimize the energy of the compounds, preprocess the target proteins and identify active pockets. MOE 2019 was then executed for molecular docking with 50 operations. The binding activity of both Triptolide and the protein was evaluated based on the binding energy magnitude. Results were visualized using PyMOL 2.6.0 and Discovery studio 2019 software.

### 2.10 Western blotting

Total cellular proteins were extracted using RIPA lysis buffer containing 1% phenylmethylsulfonyl fluoride (Beyotime Biotechnology), while cytoskeletal proteins were isolated using a subcellular protein extraction kit (GeneChem). Having calculated the loading volume, the protein sample was mixed in a 1:4 ratio with 5× Sampling Buffer (Beyotime Biotechnology), and heated to ensure complete denaturation. Sodium dodecyl-sulfate polyacrylamide electrophoresis (SDS-PAGE) gels were prepared according to the molecular weights of target proteins, and 25 µg samples were loaded for electrophoresis. Polyvinylidene fluoride (PVDF) membranes (Millipore, Burlington, MA, United States) were utilized to transfer proteins, and then blocked with 5% skimmed milk following the transfer. Primary antibodies were applied in accordance with manufacturer guidelines, and the membranes were incubated overnight at 4 °C with gentle shaking. Subsequently, the membranes were incubated with diluted secondary antibodies at room temperature for 2 h was used for imaging. The membrane, completely immersed in the luminescent reagent (ECL Chemiluminescence Kit, New Cell & Molecular Biotech, Suzhou, China), was placed into a Molecular Imager^®^ ChemiDoc™ XRS + gel imaging system (Bio-Rad, Hercules, CA, United States) for imaging. Resulting images were processed and quantitatively analyzed using ImageJ software (National Institutes of Health, Bethesda, MD, United States).

### 2.11 Drug affinity responsive target stability (DARTS)

The cultured cells were washed 2–3 times with PBS. Pre-cooled M-PER lysis buffer was added to harvest the cells into pre-chilled centrifuge tubes. After thorough mixing, the samples were incubated on ice for 10 min, followed by centrifugation at 18,000 × g for 10 min at 4°C. A 600 μL aliquot of the supernatant was transferred to a fresh centrifuge tube, and 66 μL of 10× TNC buffer was added for homogenization. Protein concentration was quantified using the BCA assay. To 297 μL of the protein sample, 3 μL of 100× small-molecule drug and 3 μL of DMSO were added, followed by incubation at room temperature for 1 h. The mixture was aliquoted equally into six centrifuge tubes (50 μL per tube), with one tube designated as the non-digested control. The remaining tubes were treated with protease solutions at dilution ratios of 1:200, 1:1000, and 1:2000 (duplicates per ratio) for 30 min at room temperature. After digestion, 3 μL of pre-cooled 20× protease inhibitor was added to each tube, mixed thoroughly, and placed on ice. The samples were subsequently subjected to Western blotting analysis.

### 2.12 Cellular thermal shift assay (CESTA)

Following trypsin digestion and centrifugation, the cells were washed twice with pre-cooled PBS. The cell pellets were lysed on ice for 30 min using RIPA lysis buffer (Beyotime Biotechnology) containing 1% PMSF. The lysates were centrifuged at 14,000 × g for 20 min at 4°C to collect soluble proteins from the supernatant. Protein concentration was determined via the BCA assay and adjusted to 2 μg/μL. Equal amounts of protein samples (50 μg) were incubated with 10 μM triptolide or an equivalent volume of DMSO (vehicle control) at 37°C for 2 h to ensure sufficient drug-target binding. The incubated protein samples were aliquoted into PCR tubes and subjected to thermal denaturation at gradient temperatures (45°C, 48°C, 51°C, 54°C, 57°C, 60°C, 63°C) for 3 min each. The denaturation process was immediately terminated by placing the samples on ice for 5 min. The denatured samples were analyzed by Western blotting to detect specific protein bands.

### 2.13 Molecular dynamics simulations

Molecular dynamics simulations were performed using Gromacs 2022.3 version software. (Van Der Spoel et al., 2005; Abraham et al., 2015). Small molecule preprocessing was performed using AmberTools22 to apply the GAFF force field, while Gaussian 16W was used for hydrogenation and calculation of RESP potentials. The simulation conditions were maintained at a static temperature of 300K and atmospheric pressure (1 Bar). The Amber99sb-ildn force field was employed, with water molecules as the solvent (Tip3p water model), and the simulation system’s total charge was neutralized by adding an appropriate number of Na + ions. The molecular dynamics simulation system was first minimized using the steepest descent method, followed by 100,000 steps of isothermal isovolumic (NVT) equilibrium and isothermal isobaric (NPT) equilibrium, each with a coupling constant of 0.1 ps and a duration of 100 ps. Finally, a free molecular dynamics simulation was run for a total of 5,000,000 steps of 2 fs duration, equivalent to 100 ns. After the completion of the simulation, the trajectories were analyzed using the software’s built-in tools to calculate the root mean square deviation (RMSD), root mean square rise and fall (RMSF), and protein radius of gyration (Rg) for each amino acid trajectory, combined with the free energy (MMGBSA), and the free energy topography.

### 2.14 Statistical analysis

Each experiment was repeated at least three times. Data were analyzed using one-way analysis of variance with SPSS 16.0 (SPSS Inc., Chicago, IL). Data are shown as means ± standard deviations. P value <0.05 indicates statistically significant differences.

## 3 Results

### 3.1 TPL inhibits the migration and invasion ability of RA-FLS

**FIGURE 1:**
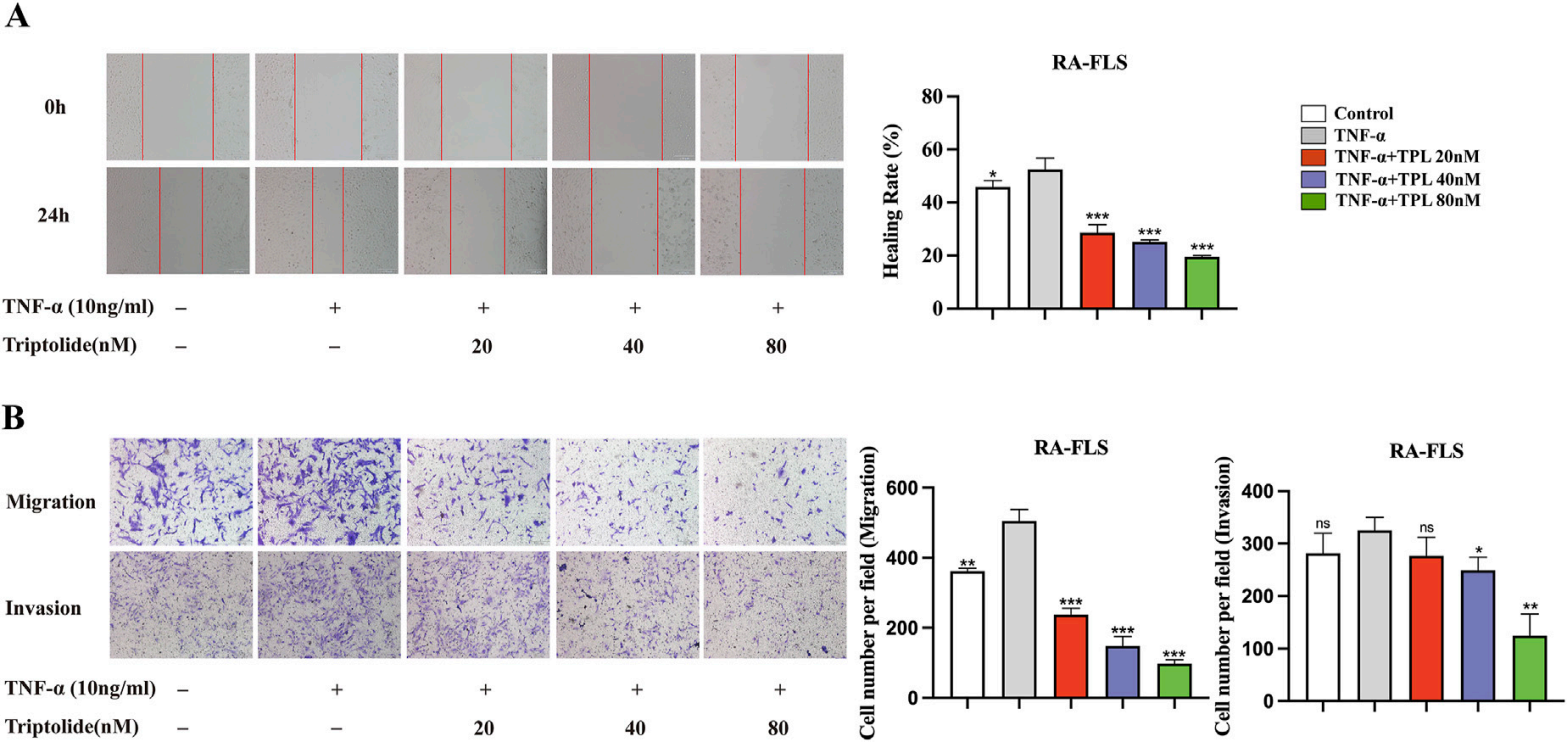
Triptolide inhibits the migration and invasion ability of rheumatoid arthritis human fibroblast-like synoviocytes. **(A)** Wound healing assay of RA-FLS under the intervention of different concentrations of TPL. **(B)** Migration and invasion transwell assay of RA-FLS in each group. *P < 0.05, **P < 0.01, and ***P < 0.001. ns: no statistical significance, compared to TNF-α group.

Migration and invasion of synovial fibroblasts were assessed using wound healing and Transwell assays to investigate the inhibitory effect of TPL. The wound healing assay results indicated that TNF-α stimulation significantly enhanced the healing ability of RA-FLS cells compared to the control group. However, TPL-treated RA-FLS cell group exhibited decreased wound area and significantly reduced cell healing ability compared to the TNF-α group. (Figure 1A). The Transwell assay results showed a significant increase in the number of cell-permeable membranes of RA-FLS cells stimulated by TNF-α compared to the control group. Additionally, under TNF-α stimulation, the number of cell-permeable membranes in the TPL-treated RA-FLS cell group was significantly reduced, inhibiting cell invasion and migration. These functional experiments demonstrated that TPL inhibited the migration and invasion of RA-FLS in a dose-dependent manner *in vitro* (Figure 1B). Microscopic images of RA-FLS were obtained at ×200 magnification.

### 3.2 Dynamic image of TPL inhibiting the cell mobility of RA-FLS

**FIGURE 2:**
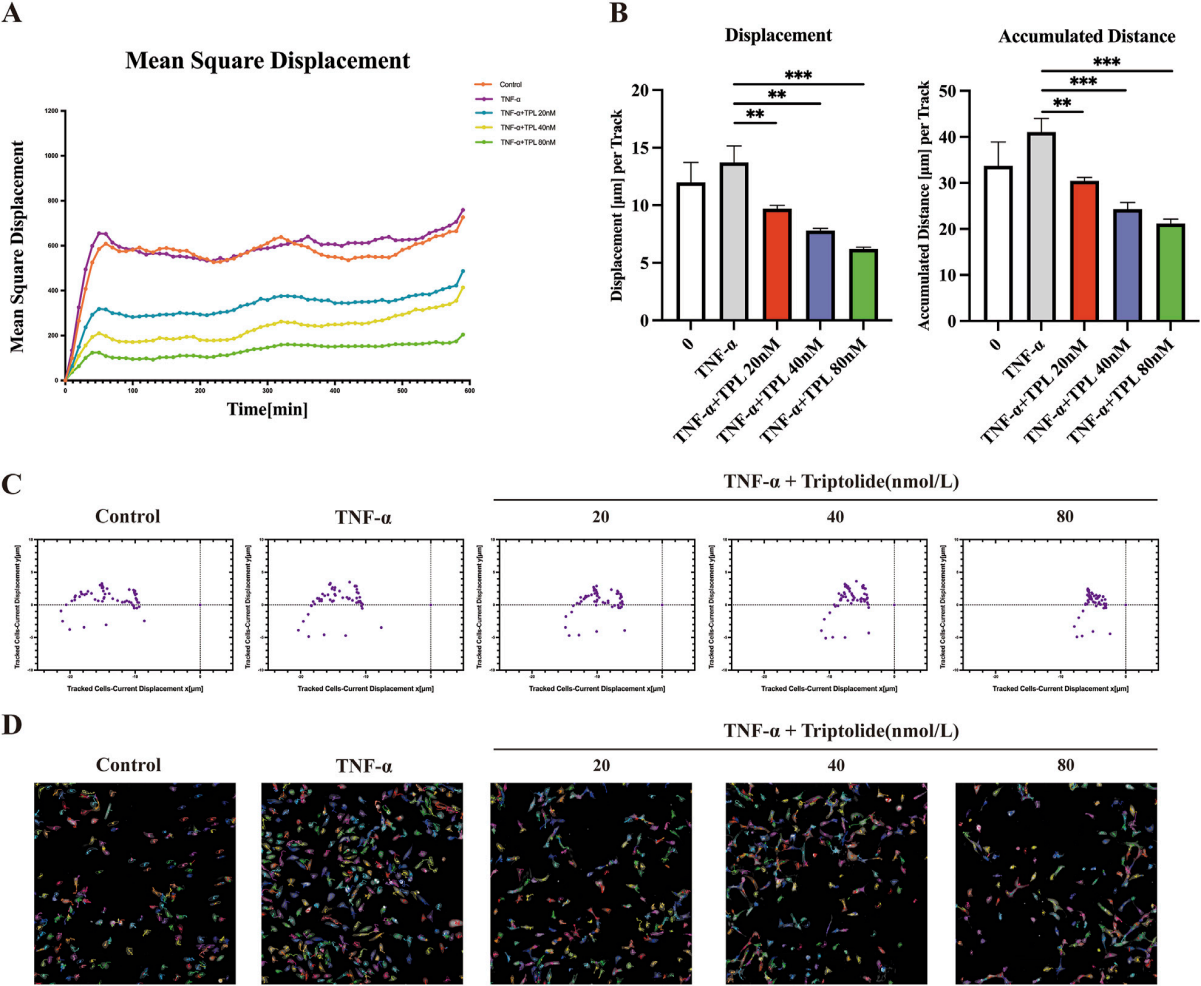
Triptolide inhibits cell mobility of rheumatoid arthritis human fibroblast-like synoviocytes. **(A)** Mean square displacement vs observation time from well level data. **(B)** Cumulative distance and displacement of RA-FLS cell movement in each group. **(C)** Visualization of cell displacement. Current displacement Y was plotted against current displacement X. Each point corresponds to the displacement of a cell at a given time point. Mean square displacement: squared norm of a vector from the first point to the current observation point; averaged over all cells per well. Current displacement X (mm): X component of a vector from the first point to the current observation point; averaged over all cells per well. Current displacement Y (mm): Y component of a vector from the first point to the current observation point; averaged over all cells per well. **(D)** PerkinElmer Operetta CLS High-content Imaging System was used to observe the effects of TPL intervention on cell mobility over 12 h, imaged with a ×20 objective in the DPC channel. Cells were identified using the Find Cells module, and migration was monitored using the Track Objects module. *P < 0.05, **P < 0.01, and ***P < 0.001.

To further investigate cell movement under TPL intervention, we utilized the Operetta CLS High-content Imaging System Analysis to visualize cell movement. The results showed that RA-FLS exhibited a decreasing trend in mean-square displacement (vector leveling method from the first to current displacement observation point, averaged over all cells in the well) under different TPL concentrations as observation time increased (Figure 2A). Displacement amount and accumulative distance traveled by FLS were significantly reduced by TPL stimulation (Figure 2B). After approximately 10 h of continuous observation, the movement trajectory of FLS was visualized, indicating that TPL could inhibit FLS migration (Figure 2C). The digital phase contrast (DPC) method was utilized for cell segmentation, and unlabeled cells were tracked and imaged in the DPC channel (Figure 2D). As the concentration of TPL increased, cell movement trajectories were shortened, indicating inhibition of cell motility.

### 3.3 Effects of TPL on cytoskeletal remodeling in RA-FLS

**FIGURE 3:**
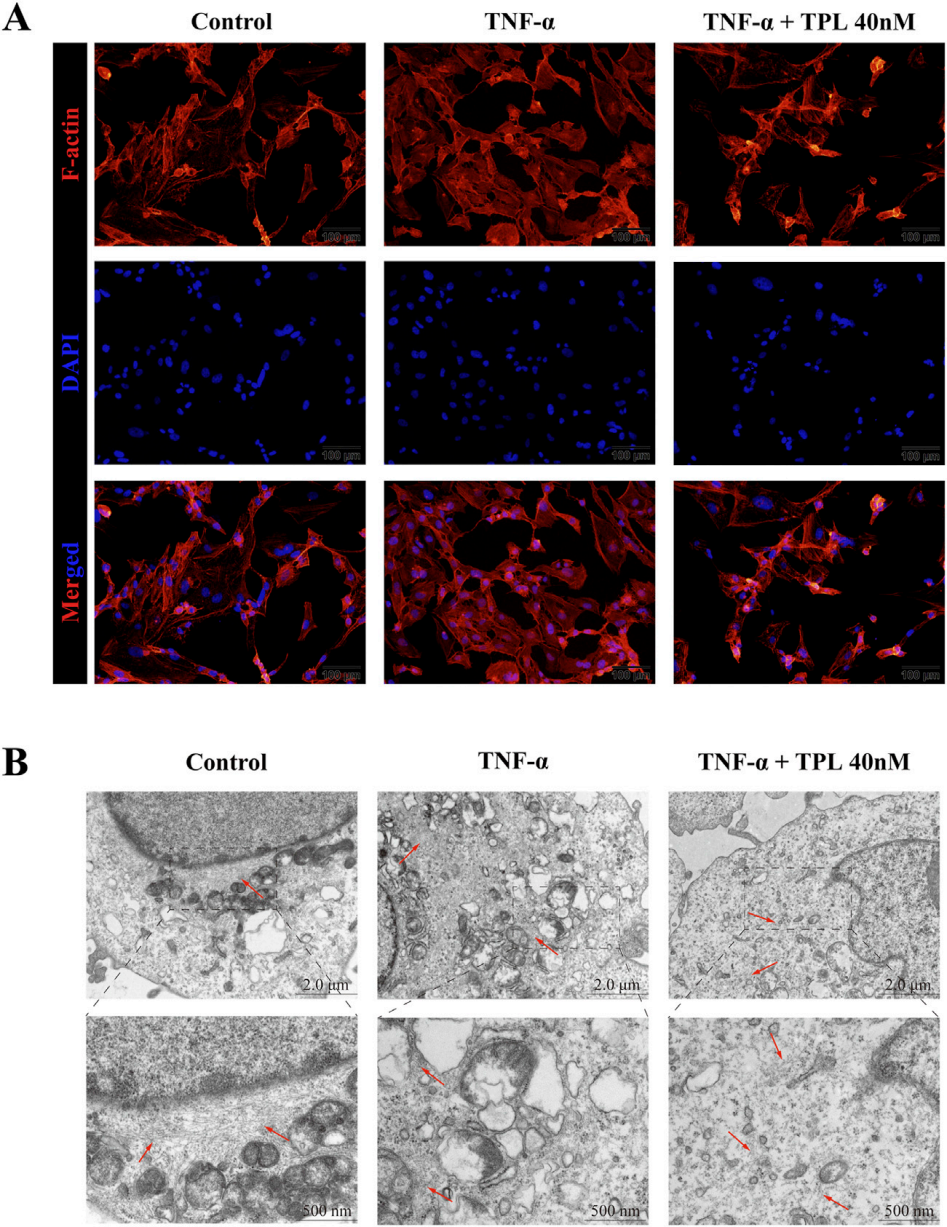
The actin cytoskeleton remodeling of rheumatoid arthritis human fibroblast-like synoviocytes is affected by Triptolide. **(A)** Cytoskeletal staining was used to observe the fluorescence and morphological changes of F-actin in RA-FLS. Compared with the TNF-α group, F-actin fluorescence was diminished and the number of pseudopods was relatively reduced in the TPL group. **(B)** Transmission electron microscopy (TEM) was used to observe microfilament changes in RA-FLS. The number of microfilaments in the TPL group was less than that in the control and TNF-α groups and the arrangement of microfilaments tended to be disordered. Red arrows indicate cellular microfilaments.

To investigate the cytoskeleton remodeling effect of TPL on RA-FL, we performed cytoskeleton staining experiments with phalloidin. TNF-α stimulation resulted in darker staining, intact cytoskeletal structure, and increased pseudopod formation. After 24 h of 40 nM TPL treatment, cytoskeleton staining was lighter, the number of pseudopods was reduced, and the skeleton structure was broken (Figure 3A). TEM observation of microfilament and microtubule distribution further confirmed these findings. The control group showed normal microfilament and microtubule number and distribution, while TNF-α stimulation increased the number and aggregated them into bundles. TPL treatment resulted in sparse, scattered microfilament and microtubule distribution (Figure 3B). Together, these results indicated that TPL could destabilize the RA-FLS cytoskeleton.

### 3.4 Effect of TPL on the expression of epithelial–mesenchymal transition (EMT)-Related and matrix metalloproteinases (MMPs) proteins in RA-FLS

**FIGURE 4:**
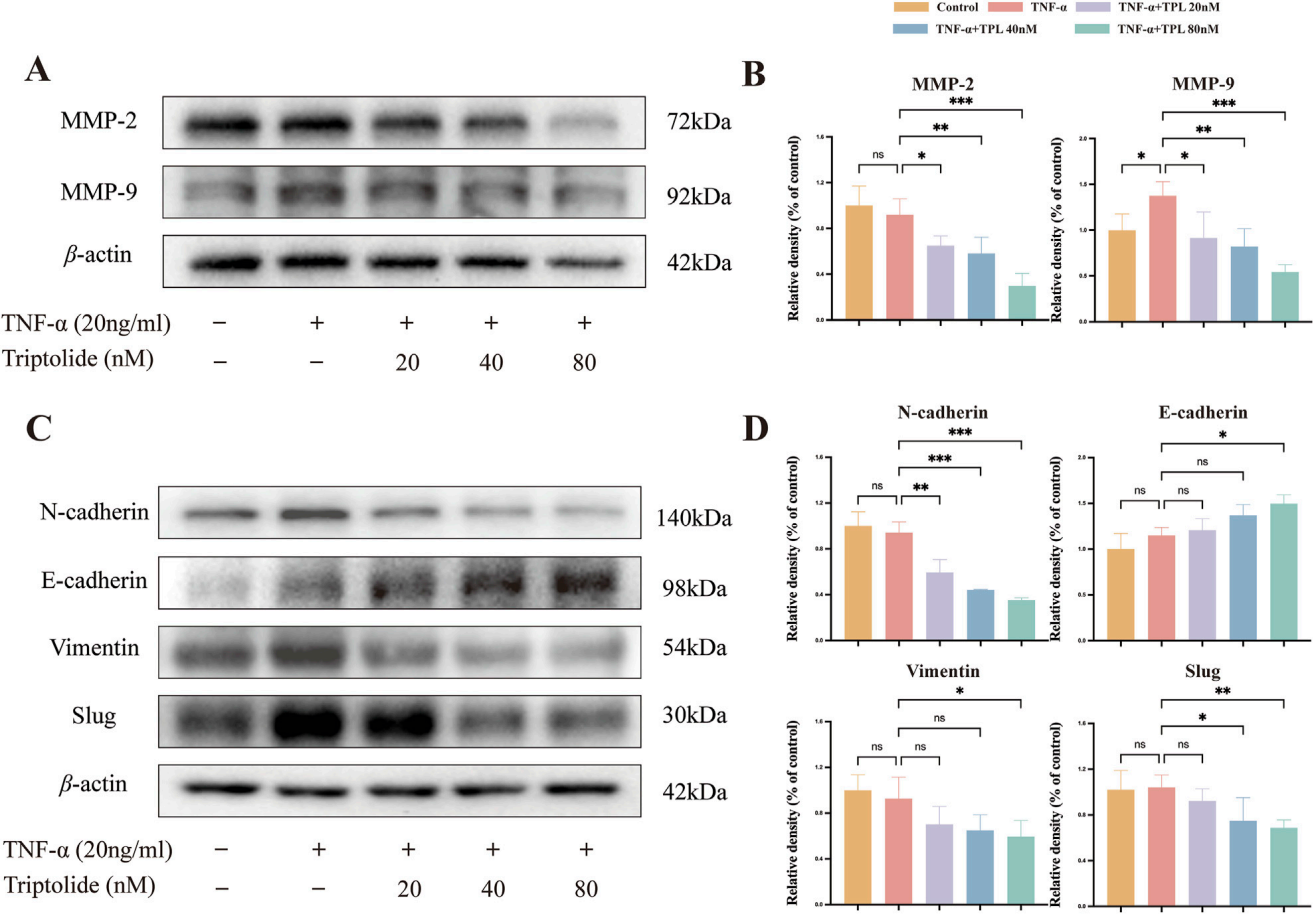
Epithelial-mesenchymal transition (EMT) process and ix Metalloproteinases (MMPs) of rheumatoid arthritis human fibroblast-like synoviocytes were inhibited by triptolide. **(A, C)** Western blotting was used to detect the expression levels of proteins involved in EMT signaling pathway (E-cadherin, N-cadherin, Vimentin, Slug)and matrix metalloproteinases (MMP9, MMP2) in RA-FLS. **(B, D)** The panel shows the quantified statistical plot drawn after statistical analysis.

Epithelial-mesenchymal transition is a fundamental biological process that plays a significant role in RA-FLS migration and invasion (Ma et al., 2021). After 24-h TPL treatment, total cellular proteins were extracted and analyzed for proteins associated with epithelial-mesenchymal transition and metastasis. TPL significantly increased the expression of E-cadherin, an epithelial marker protein present in both epithelial and metastatic cells, compared to the group stimulated with TNF-α only. In addition, TPL showed a substantial inhibition of the expression of mesenchymal cell marker proteins, including N-cadherin and Vimentin (Figures 4C, D). Moreover, the expression levels of EMT-related protein Slug and metastasis-related proteins MMP-2 and MMP-9 were significantly reduced (Figures 4A, B). These results indicate that TPL suppresses EMT and impedes the invasion and metastasis of RA-FLS.

### 3.5 Cyber-pharmacology predicts Rho-GTPases the target of TPL for cytoskeleton remodeling

**FIGURE 5:**
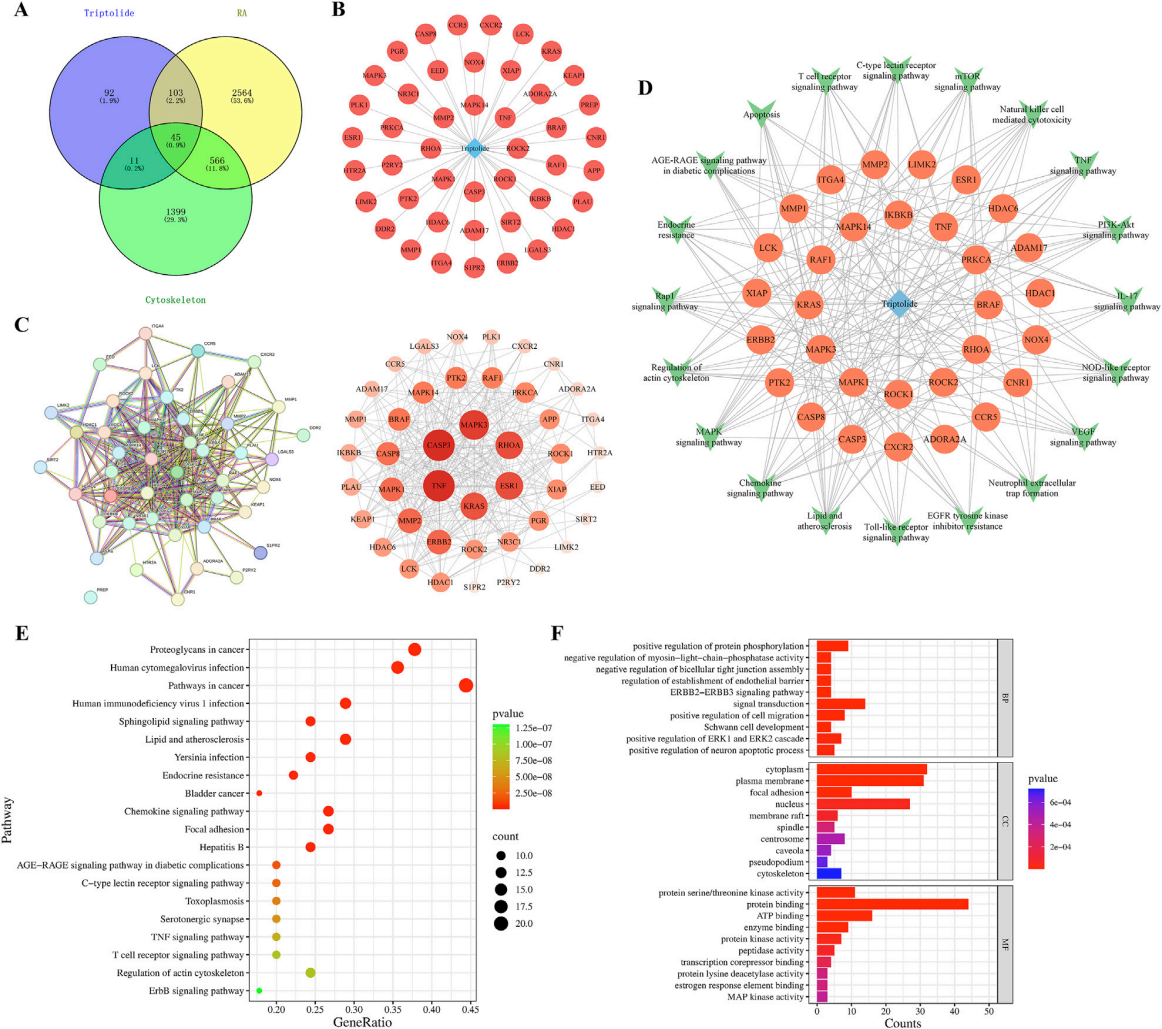
Prediction of Triptolide’s targets through cytoskeleton intervention in rheumatoid arthritis by network pharmacology. **(A)** Intersection target Venn diagram of Triptolide, cytoskeleton, and rheumatoid arthritis. **(B)** Network diagram of compound and intersecting targets. **(C)** PPI network for protein interaction analysis of cross targets. **(D)** The compound target pathway network diagram corresponding to the pathways to be visualized and the enriched targets. **(E)** KEGG pathway enrichment analysis including 20 significant enrichment signaling pathways. **(F)** GO enrichment analysis, including 10 significant enrichment terms of Biological Process (BP), Cellular Component (CC) and Molecular Function (MF).

Through searching relevant databases, 251 triptolide drug targets, 3,278 rheumatoid arthritis disease targets, and 2,021cytoskeleton-related targets were obtained. After merging, a total of 45 targets related to TPL intervention in RA via cytoskeleton were screened (Figures 5A, B). The 45 intersections of TPL were entered into the String database to obtain protein-protein interaction (PPI) network maps, which were then visualized in Cytoscape 3.9.0. The relatively important target genes were located in the center region of this network, including TNF, KRAS, ESR1, RHOA, MAPK3, and CASP3 (Figure 5C). The network diagram, a representation of the compound-target-pathway, network diagram was developed, and topology analysis provided relevant data. Prominent pathways included: the actin cytoskeleton signaling pathway, MAPK signaling pathway, TNF signaling pathway, and apoptosis signaling pathway (Figure 5D). GO and KEGG enrichment analyses were performed on the 45 cross-targets of TPL for the treatment of RA via the cytoskeleton. KEGG enrichment analysis revealed 20 relevant signaling pathways, with actin cytoskeleton-related pathways among them (Figure 5E). GO enrichment showed 254 results related to biological processes, 57 related to molecular functions, and 49 related to cellular components. The biological processes were mainly related to protein phosphorylation and myosin light chain phosphatase activity (Figure 5F).

### 3.6 TPL inhibits the RhoA/Rho-associated kinase signaling pathway of RA-FLS *in vivo*

**FIGURE 6:**
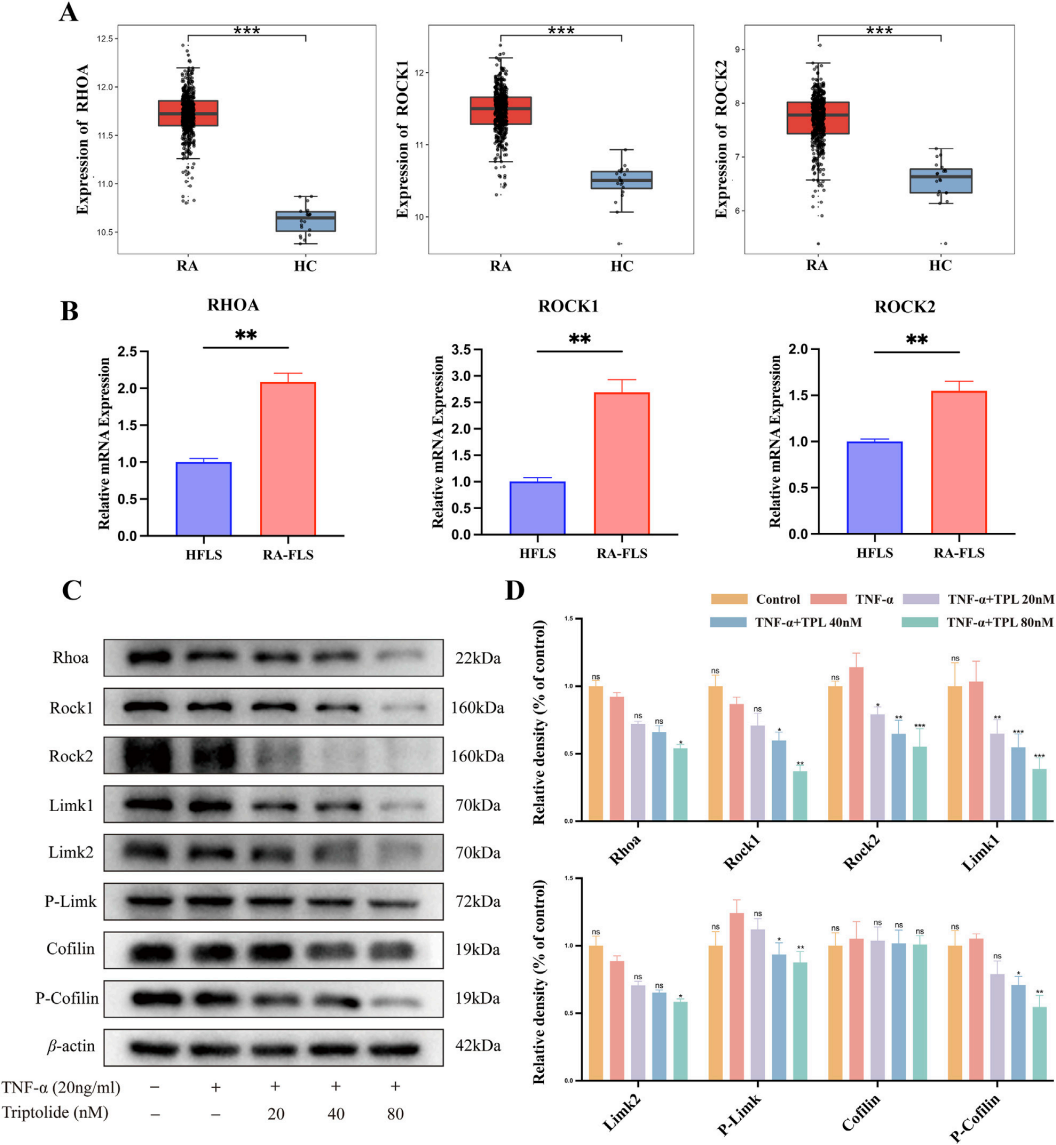
Triptolide inhibits RhoA/Rho-associated kinase signaling pathway in rheumatoid arthritis synovial cells *in vitro*. **(A)** Transcriptomics reveals high expression of RHOA, ROCK1, and ROCK2 in peripheral blood of rheumatoid arthritis patients compared to healthy controls. **(B)** The mRNA expression levels of RHOA, ROCK1, and ROCK2 in RA-FLS were significantly higher than those in HFLS. **(C)** Western blotting was used to detect the expression levels of proteins involved in RhoA/Rho-associated kinase signaling pathway (RhoA, Rock1, Rock2, Limk1, Limk2, Phospho-Limk, Cofilin, Phospho-Cofilin) in RA-FLS. **(D)** The right panel shows the quantified statistical plot drawn after statistical analysis.

The Rheumatoid Arthritis Bioinformatics Center (RABC, http://www.onethird-lab.com/RABC/, RABCID: RABC39) was searched for rheumatoid arthritis transcriptomics data revealed that RHOA, ROCK1, and ROCK2 expression was higher in rheumatoid arthritis patients than healthy controls (Figure 6A). We confirmed this variability in RA-FLS and HFLS using qRT-PCR (Figure 6B). Under different TPL concentrations, RhoA protein expression significantly differed from the TNF-α group only under high concentration treatment. TPL exposure decreased Rock protein expression, suggesting TPL inhibits Rock protein expression. Similar to Rock, TPL inhibited Limk protein expression and phosphorylation. Cofilin protein expression was not significantly different from controls at all concentrations, but phosphorylated Cofilin protein expression decreased significantly with increasing TPL, suggesting TPL inhibits Cofilin phosphorylation (Figures 6C, D).

### 3.7 Molecular docking predicts the binding ability of TPL to RhoA, Rock1, and Rock2

**FIGURE 7:**
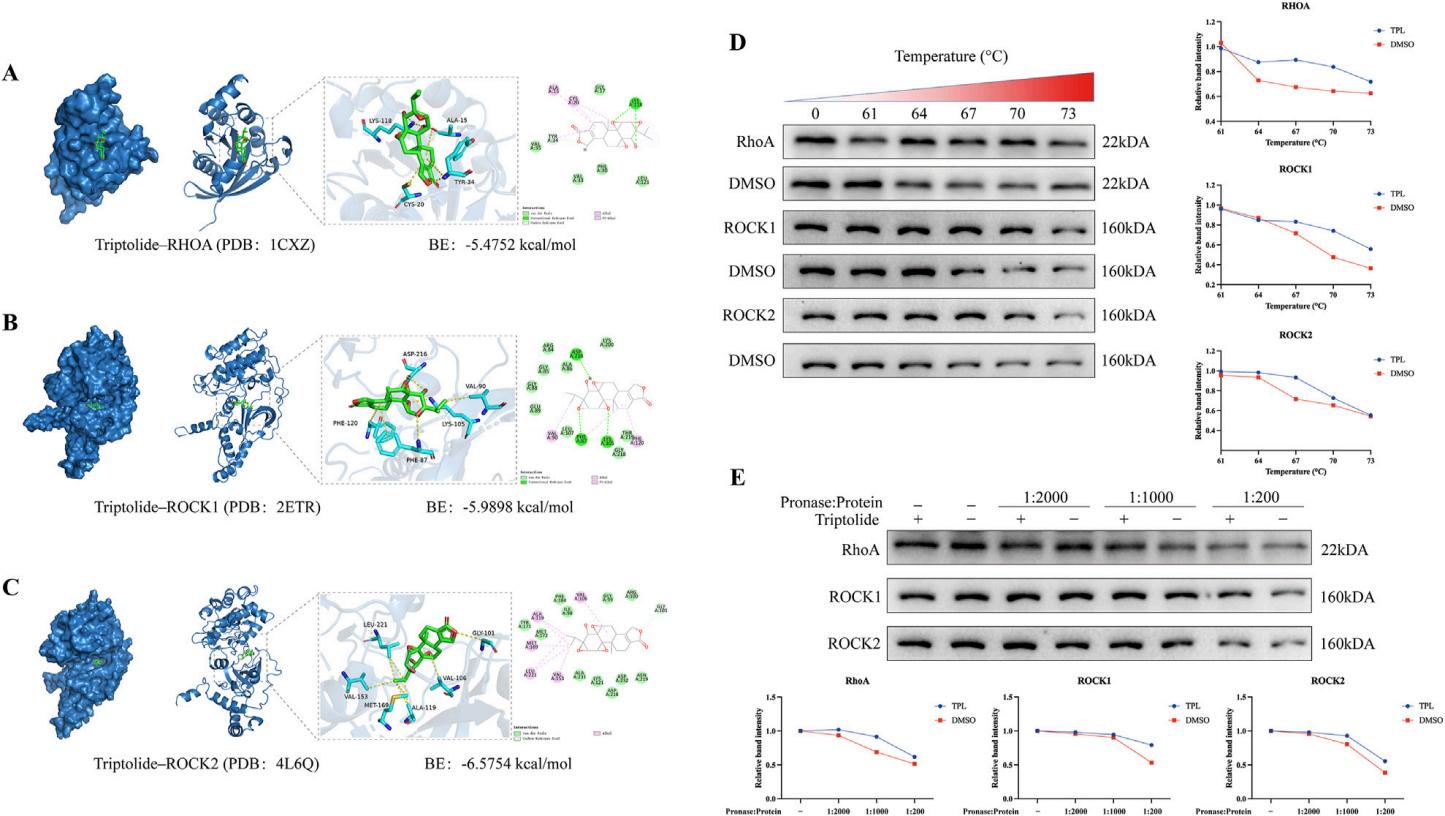
Molecular docking predicts favorable binding activity of Triptolide to RhoA, Rock1, and Rock2 proteins. **(A)** Molecular docking diagram of Triptolide and RhoA protein. **(B)** Molecular docking diagram of Triptolide and Rock1 protein. **(C)** Molecular docking diagram of Triptolide and Rock2 protein. The left image shows the binding pocket, while the right panel displays the interaction relationship from a 2D perspective. **(D)** Triptolide treatment enhances the thermal stability of RhoA, ROCK1and ROCK2 in cell lysates as determined by the CETSA (n = 3). **(E)** Triptolide treatment enhances the protease stability of RhoA, ROCK1and ROCK2 in cell lysates as determined by the DARTS assay (n = 3).

To investigate the interaction of TPL with RhoA, Rock1 and Rock2 protein targets, molecular docking was conducted using MOE 2019 software. According to the binding domains, TPL can enter the binding domains of RhoA, Rock1 and Rock2 target proteins. The molecular docking energies were −5.4752, −5.9898, and −6.5754 kcal/mol, respectively. In general, docking energies of less than −4.25 kcal/mol suggest some level of binding activity between two molecules, whereas energies of less than −5.0 kcal/mol indicate good binding activity. The docking energy value less than −4.25 kcal/mol suggests some binding activity, less than −5.0 kcal/mol indicates good binding activity, and less than −7.0 kcal/mol suggests strong binding activity. (Figures 7A–C). The docking results showed that TPL has good binding activity to RhoA, Rock1, and Rock2 proteins. DARTS and CESTA have been validated *in vitro* to enhance the thermal stability and protein sensitivity of RhoA, ROCK1, and ROCK2 in RA-FLS. Compared with the DMSO group, TPL treatment significantly improved the thermal stability of RhoA, ROCK1, and ROCK2 in different ranges from 64°C to 73 °C (Figure 7D). The presence of TPL significantly reduces the protein hydrolysis of RhoA, ROCK1, and ROCK2 by medium to high concentration proteases, suggesting possible target proteins for TPL binding in RA-FLS (Figure 7E).

### 3.8 Validation of the binding stability and intermolecular interactions of TPL with RhoA, Rock1 and Rock2

**FIGURE 8:**
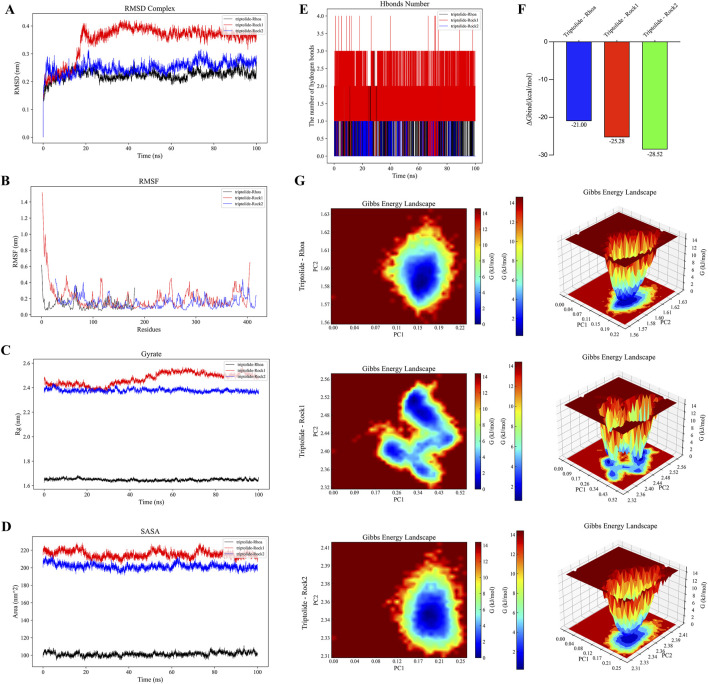
Molecular dynamics simulations with Gromacs further revealed the binding and interactions between TPL and RhoA/Rho-associated kinase. **(A)** RMSD chart of the complex of TPL bound to RhoA, Rock1, and Rock2. **(B)** RMSF chart of the complex of TPL bound to RhoA, Rock1, and Rock2. **(C)** Gyrate chart of the complex of TPL bound to RhoA, Rock1, and Rock2. **(D)** SASA chart of the complex of TPL bound to RhoA, Rock1, and Rock2. **(E)** Hbonds number of the complex of TPL bound to RhoA, Rock1, and Rock2. **(F)** ΔGbind of the complex of TPL bound to RhoA, Rock1, and Rock2. **(G)** The free energy landscape is reflected by the root-mean-square deviation (RMSD) and radius of gyration; more stable conformations generally correspond to lower free energy regions (blue regions), while less stable conformations correspond to higher free energy regions (red regions).

To investigate TPL-RhoA, Rock1, Rock2 binding, we performed molecular dynamics simulations using Gromacs. We calculated and plotted RMSD, RMSF, Gyrate, and SASA values between TPL and RhoA, Rock1, Rock2. The results demonstrate that the RMSD between TPL and RhoA, Rock2 is relatively stable, while the RMSD between TPL and Rock1 is large at the beginning of the simulation for both protein-protein and small molecule interactions, but it stabilizes after 20 ns. Overall, RMSD values of TPL with RhoA, Rock1, Rock2 are maintained in small range, indicating stable protein-small molecule binding (Figure 8A). The RMSF values of proteins are smaller in bound portion and larger in unbound portion, indicating that small molecule binding affects protein stability and flexibility of amino acids at TPL binding position with Rock1 is more variable compared to RhoA, Rock2 (Figure 8B). The Gyrate value of TPL with RhoA is significantly lower than Rock1, Rock2, indicating more compact TPL-RhoA binding. And Gyrate value fluctuates more during TPL-Rock1 binding, suggesting dynamic changes in protein structure (Figure 8C). The SASA values of TPL bound to RhoA, Rock1, Rock2 remain stable during simulation with overall unchanged trend suggesting little effect on protein surface characteristics and stability after binding (Figure 8D). Hydrogen bonds are formed between the protein and small molecule during simulation, mainly consisting of hydrogen bonds between certain key protein residues and significant groups in the small molecule (Figure 8E). The binding free energies (ΔGbind) of the three protein-ligand complexes are further calculated using MM/GBSA method. Lower ΔGbind indicates stronger receptor-ligand binding affinity (Figure 8F). As shown, the ΔGbind rankings of the three complexes are TPL-RhoA (−21.00 kcal/mol) < TPL-Rock1 (−25.28 kcal/mol) < TPL-Rock2 (−28.52 kcal/mol), consistent with molecular docking results (Figures 7A–C).

We plot Free Energy Landscape using both RMSD and R(g) (radius gyration) metrics to reflect interactions and energy distribution between molecules in the binding system. The results show a stable conformation in the TPL-RhoA, Rock2 binding system, while the TPL-Rock1 system consists of several near-equilibrium conformations, suggesting possible structural changes during TPL-Rock1 binding (Figure 8G).

## 4 Discussion

Rheumatoid arthritis (RA) is a chronic autoimmune disorder characterized by inflammatory synovitis, hyperplasia, and destructive alterations in articular synovium, ultimately leading to joint cartilage and bone damage. Current clinical management incorporates traditional Chinese medicinal extracts such as Tripterygium glycosides tablets and total glucosides of peony into first- and second-line therapies, alongside conventional treatments including non-steroidal anti-inflammatory drugs (NSAIDs), disease-modifying antirheumatic drugs (DMARDs), glucocorticoids, and biologics like TNF-α inhibitor adalimumab (Herenius et al., 2011), CD20-targeting rituximab (Humby et al., 2021), and JAK inhibitor baricitinib (Lopez-Romero et al., 2022). Despite demonstrated clinical efficacy, the precise regulatory mechanisms of these therapeutic regimens on the invasive phenotype of rheumatoid fibroblast-like synoviocytes (RA-FLSs) remain incompletely elucidated. Triptolide (TPL), an epoxidized diterpene lactone and principal active component of Tripterygium glycosides, has been previously reported to suppress RA-FLS proliferation while enhancing chondrocyte viability and secretory function (Zhang et al., 2024a; Li et al., 2022). This study elucidates a novel mechanism whereby TPL coordinates cytoskeletal reorganization with epithelial-mesenchymal transition (EMT) inhibition through modulation of the RhoA/Rho-associated kinase (ROCK) signaling pathway, providing molecular insights into its clinical effects.

Convergent multi-omics analyses established the central role of RhoA/ROCK signaling in cellular motility and cytoskeletal remodeling (McBeath et al., 2004). RhoA, a small GTPase belonging to the Ras superfamily, cycles between inactive (GDP-bound) and active (GTP-bound) states to regulate diverse cellular processes including cytoskeletal rearrangement, cell migration, phagocytosis, vesicular trafficking, transcriptional regulation, and cellular growth (van Haren et al., 2014). ROCK (Rho-associated coiled-coil containing kinase), a serine/threonine kinase functioning as the primary RhoA effector, mediates downstream signaling through phosphorylation of target proteins including myosin light chain (MLC), LIM kinase, intermediate filaments (vimentin, GFAP, NF), and microtubule-associated protein 2 (MAP2) (Tang Y. et al., 2018). Network pharmacology screening identified RhoA and its downstream effectors as central hubs in the “TPL-RA-cytoskeleton” interaction network, demonstrating significant crosstalk with canonical inflammatory pathways such as TNF and MAPK. Gene Ontology (GO) and KEGG enrichment analyses revealed TPL-associated biological processes primarily involving protein phosphorylation and myosin light chain phosphatase activity. Transcriptomic profiling further demonstrated aberrant RhoA pathway activation in peripheral blood and synovial cells from RA patients, with persistent hyperactivity observed in cultured RA-FLSs, suggesting its role as a molecular switch maintaining pathological synovial phenotypes.

The RhoA/ROCK axis bridges cytoskeletal dynamics and EMT progression through dual regulatory mechanisms. Cytoskeletal architecture provides the structural basis for invasive pseudopod and filopodia formation - essential cellular locomotory apparatus (Chu et al., 2022). Dynamic cytoskeletal remodeling, particularly pseudopod generation, plays pivotal roles in RA synovial cell invasion and migration (Liu et al., 2022; Laragione et al., 2018). RhoA governs migratory mechanics primarily through direct regulation of actin cytoskeletal reorganization, stress fiber formation, and focal adhesion dynamics via downstream effectors like ROCK (Liu et al., 2016). TPL-induced suppression of ROCK-LIMK-Cofilin signaling enhanced actin depolymerization, disrupting microfilament bundle orientation and effectively inhibiting pseudopod formation - the physical driver of cell migration. Concurrently, TPL attenuated EMT transcriptional reprogramming, evidenced by restored E-cadherin membrane localization and coordinated downregulation of N-cadherin/MMP-9 expression. This mechanochemical coordination enables TPL to simultaneously target both initiation (EMT) and execution (cell motility) phases of the invasive phenotype, demonstrating superior therapeutic breadth compared to single-mechanism agents. Unlike its direct involvement in cytoskeletal remodeling, RhoA/ROCK participation in EMT manifests through indirect mechanisms (Ma et al., 2019). While core EMT programs are transcriptionally driven by master regulators coordinating molecular reprogramming events like E-cadherin downregulation, RhoA primarily enhances post-EMT cellular behaviors through migration/invasion potentiation (Shan et al., 2023; Jung et al., 2019). This ancillary role modulates morphological plasticity via crosstalk with EMT-inducing signals like TGF-β (Du et al., 2021). RhoA/ROCK signaling intersects with EMT transcription regulatory factors. Studies have demonstrated that ROCK1 has been shown to phosphorylate Smad2/3, enhancing its interaction with Snail to amplify TGF-β-induced EMT (Tang L. et al., 2018). Our findings align with these reports, as triptolide treatment significantly reduced nuclear accumulation of Snail and restored membrane-associated E-cadherin, indicating that RhoA inhibition disrupts the feed-forward loop between cytoskeletal tension and EMT transcription factor activation. This dual targeting of both structural and transcriptional EMT drivers highlights triptolide’s potential as a multi-mechanistic anti-metastatic agent.

Molecular docking and dynamics simulations revealed TPL’s interaction patterns and dynamic binding characteristics with RhoA, ROCK1, and ROCK2. TPL formed hydrogen bonds and hydrophobic interactions with key residues including RhoA’s Lys118 and Cys20, while stabilizing within ROCK1/2 binding pockets through interactions with Asp216/Phe87 (ROCK1) and Val106/Ala119 (ROCK2). GROMACS-based molecular dynamics simulations, widely employed for biomolecular system analysis (Yekeen et al., 2023), demonstrated structural stability through root-mean-square deviation (RMSD) assessment (An et al., 2024), residue flexibility via root-mean-square fluctuation (RMSF) analysis (Li et al., 2024), and structural compactness using radius of gyration (Rg) measurements (Zhi et al., 2021). RhoA-TPL, ROCK1-TPL, and ROCK2-TPL complexes achieved RMSD equilibrium after 50 ns, indicating conformational convergence upon TPL binding. Notably, free energy landscape analysis revealed catalytic domain rigidification in ROCK1, potentially inhibiting phosphorylation capacity through kinase active-site conformational modulation. This mechanism aligns with observed ROCK substrate (LIMK) phosphorylation reduction in Western blotting, collectively elucidating TPL’s spatial hindrance and allosteric effects on Rho/ROCK signaling. Drug affinity responsive target stability (DARTS) and cellular thermal shift assay (CETSA) validated TPL’s direct binding: DARTS demonstrated enhanced protease resistance in TPL-treated RhoA/ROCK proteins, consistent with docking-predicted binding site distribution. CETSA revealed distinct thermal stabilization (ΔTm) between ROCK1 and ROCK2, correlating with MM/PBSA-calculated binding free energy rankings from molecular dynamics.

Notably, RhoA’s pathogenic role in RA is corroborated by *in vivo* evidence. Collagen-induced arthritis (CIA) model rats exhibited elevated synovial RhoA activation, while RhoA silencing suppressed osteoclast differentiation *in vitro* and alleviated synovial hyperplasia/bone erosion in CIA mice (Chen N. et al., 2023). TPL administration (0.1 mg/kg/day) significantly reduced synovial hyperplasia and bone erosion in CIA rats, paralleled by downregulation of rheumatoid factor, IL-17, TNF-α, IL-1β, and IL-6 (Zhang et al., 2024b). Our mechanistic findings align with these therapeutic outcomes: TPL’s competitive inhibition of RhoA-GTP binding and direct ROCK kinase activity interference likely synergistically underlie its anti-invasive effects. Future studies employing conditional RhoA knockout/overexpress models could further validate pathway necessity in TPL’s *in vivo* efficacy, advancing modernization research of traditional Chinese medicine.

In conclusion, TPL’s specific modulation of the RhoA/ROCK axis represents the core mechanism suppressing synovial cell invasiveness, elevating traditional medicine mechanism research to the novel dimension of cellular mechanoregulation. This specificity may partially explain TPL’s unique clinical efficacy profile, particularly its advantage in retarding structural joint damage. These findings not only provide precise mechanistic insights for TPL’s clinical application but also establish theoretical foundations for developing novel RA therapeutics targeting cellular motility regulation. Future studies employing conditional RhoA knockout models could further validate pathway necessity in TPL’s *in vivo* efficacy, advancing modernization research of traditional Chinese medicine.

## 5 Conclusion

In conclusion, this study demonstrates that triptolide, a small molecule herbal monomer compound, inhibits the migration and invasion of RA-FLS by modulating the RhoA/Rho-associated kinase signaling pathway and mediating cytoskeletal remodeling. Triptolide disrupted microfilament and microtubule arrangement, inhibited pseudopod formation, and suppressed the EMT process and invasive ability of RA-FLS by regulating EMT-related protein expression and matrix metalloproteinases (MMPs) levels. Triptolide stabilized interactions with RhoA, Rock1, and Rock2, resulting in more compact protein molecules, and may alter the conformation of Rock1, affecting its function. This study elucidates the pharmacological mechanism of triptolide, provides a sound theoretical and experimental foundation for further research, and offers a new theoretical basis for the clinical application of triptolide in the treatment of rheumatoid arthritis.

## Data Availability

The original contributions presented in the study are included in the article/Supplementary Material, further inquiries can be directed to the corresponding authors.
