# Supplementary material for: Exploring the mechanism of triptolide inhibiting the motility of fibroblast-like synoviocytes in rheumatoid arthritis via RhoA/Rho-associated kinase axis, based on network pharmacology, molecular docking and molecular dynamics simulations
Source: Front Pharmacol. 2025 Apr 3;16:1545514. doi: 10.3389/fphar.2025.1545514 (PMC12003270; doi:10.3389/fphar.2025.1545514)
Supplement: Supplementary file 1 [file Table1.docx]

**Supplemental Materials**

**Table S1. Sequences of the primers used for quantitative real-time PCR.**

| **Gene** | **Forward primer (5′–3′)** | **Reverse primer (5′–3′)** |
| --- | --- | --- |
| RHOA | CCAAGATGAAGCAGGAGCCG | AGCAGCTCTCGTAGCCATTT |
| ROCK1 | TGCTGGTAAGAGGGCATTGT | AAAGCATCCAATCCATCCAGC |
| ROCK2 | GAGAGCTTGCTGGATGGCT | TCTGCCTTCATCTGTAGACCTC |
| ACTIN BETA | GCACTCTTCCAGCCTTCCTTCC | GCGGATGTCCACGTCACACTTC |

**Table S2. The antibodies used in this study.**

| **Antibody** | **Company** | **Cat#** | **Dilution** |
| --- | --- | --- | --- |
| MMP-2 | ThermoFisher Scientific | PA5-85197 | 1:500 |
| MMP-9 | ThermoFisher Scientific | PA5-16509 | 1:500 |
| N-cadherin | ThermoFisher Scientific | 33-3900 | 1:500 |
| E-cadherin | ThermoFisher Scientific | 14-3249-82 | 1:250 |
| Vimentin | ThermoFisher Scientific | MA5-16409 | 1:1000 |
| Slug | Proteintech | 12129-1-AP | 1:1000 |
| RhoA | ABclonal Biotechnology | A18695 | 1:1000 |
| ROCK1 | Cell Signaling Technology | 4035 | 1:1000 |
| ROCK2 | Cell Signaling Technology | 9029 | 1:1000 |
| LIMK1 | Cell Signaling Technology | 3842 | 1:1000 |
| LIMK2 | Cell Signaling Technology | 3845 | 1:1000 |
| Phospho-LIMK1 (Thr508)/LIMK2 (Thr505) | Cell Signaling Technology | 3841 | 1:1000 |
| Cofilin | Cell Signaling Technology | 5715 | 1:1000 |
| Phospho-Cofilin (Ser3) | Cell Signaling Technology | 3313 | 1:1000 |
| Beta-Actin | Sigma | A3854 | 1:25,000 |
| Anti-rabbit IgG, HRP-linked Antibody | Cell Signaling Technology | 7074 | 1:1000 |
| Anti-mouse IgG, HRP-linked Antibody | Cell Signaling Technology | 7076 | 1:1000 |
| Anti-rat IgG, HRP-linked Antibody | Cell Signaling Technology | 7077 | 1:1000 |
